# AFITbin: a metagenomic contig binning method using aggregate *l*-mer frequency based on initial and terminal nucleotides

**DOI:** 10.1186/s12859-024-05859-7

**Published:** 2024-07-16

**Authors:** Amin Darabi, Sayeh Sobhani, Rosa Aghdam, Changiz Eslahchi

**Affiliations:** 1 Department of Computer and Data Sciences, Faculty of Mathematical Sciences, Shahid Beheshti University, Tehran, Iran; 2 School of Biological Sciences, Institute for Research in Fundamental Sciences (IPM), Tehran, Iran; 3 Wisconsin Institute for Discovery, University of Wisconsin-Madison, Madison, WI 53715 USA

**Keywords:** Metagenomic binning, Clustering, Composition vector, k-mer, Coverage vector

## Abstract

****Background**:**

Using next-generation sequencing technologies, scientists can sequence complex microbial communities directly from the environment. Significant insights into the structure, diversity, and ecology of microbial communities have resulted from the study of metagenomics. The assembly of reads into longer contigs, which are then binned into groups of contigs that correspond to different species in the metagenomic sample, is a crucial step in the analysis of metagenomics. It is necessary to organize these contigs into operational taxonomic units (OTUs) for further taxonomic profiling and functional analysis. For binning, which is synonymous with the clustering of OTUs, the tetra-nucleotide frequency (TNF) is typically utilized as a compositional feature for each OTU.

****Results**:**

In this paper, we present AFIT, a new *l*-mer statistic vector for each contig, and AFITBin, a novel method for metagenomic binning based on AFIT and a matrix factorization method. To evaluate the performance of the AFIT vector, the t-SNE algorithm is used to compare species clustering based on AFIT and TNF information. In addition, the efficacy of AFITBin is demonstrated on both simulated and real datasets in comparison to state-of-the-art binning methods such as MetaBAT 2, MaxBin 2.0, CONCOT, MetaCon, SolidBin, BusyBee Web, and MetaBinner. To further analyze the performance of the purposed AFIT vector, we compare the barcodes of the AFIT vector and the TNF vector.

****Conclusion**:**

The results demonstrate that AFITBin shows superior performance in taxonomic identification compared to existing methods, leveraging the AFIT vector for improved results in metagenomic binning. This approach holds promise for advancing the analysis of metagenomic data, providing more reliable insights into microbial community composition and function.

****Availability**:**

A python package is available at: https://github.com/SayehSobhani/AFITBin.

**Supplementary Information:**

The online version contains supplementary material available at 10.1186/s12859-024-05859-7.

## Introduction

Metagenomics is a fascinating field of study that investigates the genetic components found in environmental samples containing diverse microbial communities. Due to advancements in sequencing technologies, it has become more accessible and affordable to sequence microbes extracted from these environmental samples directly [1]. The process of obtaining genetic data involves simple steps of sampling, sequencing, and analysis. These samples contain a diverse community of microbes that resemble a laboratory microbe colony. However, in the study of metagenomics, genetic information is obtained by taking samples from environments such as the intestine or soil, which can consist of hundreds or thousands of unknown species [2].

After the removal of impurities from these samples, filtering, isolating DNA strands, and fragmenting them, these samples are sequenced into reads [3, 4]. Metagenomic binning can be performed on reads before assembling reads into contigs. However, since the reads are significantly shorter sequences, binning is typically performed after the contigs have been constructed. These obtained contigs belong to different microbes. Therefore, for future research in metagenomics, algorithms are required to classify these contigs so that contigs related to the same organism are placed in the same class. Considering that the samples in this study may contain unknown microbial species, an unsupervised clustering method is allowed [5, 6].

Clustering, which is a major component of metagenomic binning, is the process of grouping contigs, scaffolds, or genes according to their genetic characteristics, such as oligonucleotide frequency (referred to as *l*-mers) or coverage. The three types of current approaches for retrieving bins from metagenomic assemblies are: (i) nucleotide composition-based, (ii) differential abundance-based, and (iii) nucleotide composition and abundance-based [7–9].

Composition-based approaches rely on variations in oligonucleotide frequency, specifically tetra-nucleotide frequency (TNF). In contrast, differential abundance-based approaches rely on the coverage of contigs across diverse samples in which the abundance of organisms varies. Composition and abundance-based methods focus on making a combined distance matrix by combining analyses based on nucleotide composition and differential abundance [10–12]. Composition-based approaches employ the notion that each taxonomic unit (species, genus, etc.) has a unique nucleotide composition and conducts binning by comparing nucleotide content, principally oligonucleotide frequency, and guanine-cytosine (*GC*) content [8]. The majority of composition-based approaches have been applied to communities with genotypes exhibiting distinctive nucleotide composition patterns, including low *GC* content and stable oligonucleotide frequency [13]. In order to make the operation more computationally feasible, the sequence composition information is translated into numerical feature vectors. The most prevalent attributes are the normalized frequencies of oligonucleotides of a particular length. According to studies, the frequency of oligonucleotides varies between and within species. The TNF vector is one of the most common types of oligonucleotide frequencies. TNF is a 256-dimensional vector that represents the frequency of all 4-mers. Additionally, the *GC* content is used, as studies have shown that *GC* content varies between species [14]. Even though it has not been conclusively demonstrated, it is likely that this method will fail in communities with diverse oligonucleotide compositions [7].

Abundance-based approaches assume that either the distribution of sequenced reads in a single sample follows the Lander-Waterman model [15] or that the coverage profiles of contigs from the same genomes should be highly correlated across multiple samples [6, 8, 16, 17].

Methods based on composition and abundance combine the two previously mentioned approaches into one. It has been demonstrated that by combining composition and coverage information, which indicates species abundance, additional information can be extracted from metagenomic data, resulting in more accurate binning. Coverage information is computed by a contig’s coverage, which is the average number of reads per base from the sample within the contig. Expectation-maximization (EM) algorithms, probabilistic models, and principal component analysis (PCA) are utilized by these methods [18]. CONCOCT [19] uses PCA to reduce dimensionality and the Gaussian mixture model to cluster contigs into bins. MetaCon [20] discovers various distributions of *l*-mers based on the probabilities of *l*-mers in each contig; it uses the information contained in long contigs to guide the formation of clusters. MaxBin 2.0 [21] employs the Lander-Waterman model [15] and the EM algorithm to perform iterative clustering. Based on the frequency and abundance of *l*-mers, MetaBAT 2 [22] computes probabilistic distances between pairs of contigs. SolidBin [23] utilizes spectral clustering coupled with further biological information using a semi-supervised approach. BusyBee Web [24] bins contigs utilizing a bootstrapped supervised binning method. This binning method is for contigs that are 500 bp or longer by default. Due to restricted computing resources and the fact that BusyBee Web is a web-based application, specific limits on the size of the input data have been imposed. MetaBinner [25] employs single-copy gene data for k-means clustering algorithm to produce distinct component binning outcomes. Subsequently, it combines the outcomes of component binning using a two-stage ensemble strategy based on MetaWRAP [26] and UniteM (https://github.com/dparks1134/UniteM). Some of these above-mentioned methods, however, remove small contigs (e.g., less than 1000bp) since the composition and coverage properties are not reliable for short contigs. Also, most binning methods depend on similarity metrics between contigs based on k-mers frequency distributions.

In this paper, we present AFITBin, a novel method for contig binning that can bin millions of contigs derived from numerous samples. We demonstrated that AFITBin generates high-quality bins by employing contig coverage and a newly proposed composition vector that calculates the repetition frequency of substrings based on their initial and terminal base pairs. Similar to MetaCon, AFITBin clusters contigs in two stages: first, by eliminating short contigs and creating clusters, and subsequently, by assigning short contigs to their clusters. Then, we compared AFITBin’s performance to the methods mentioned earlier.

## Method

### AFIT: a novel composition vector

**Fig. 1:**
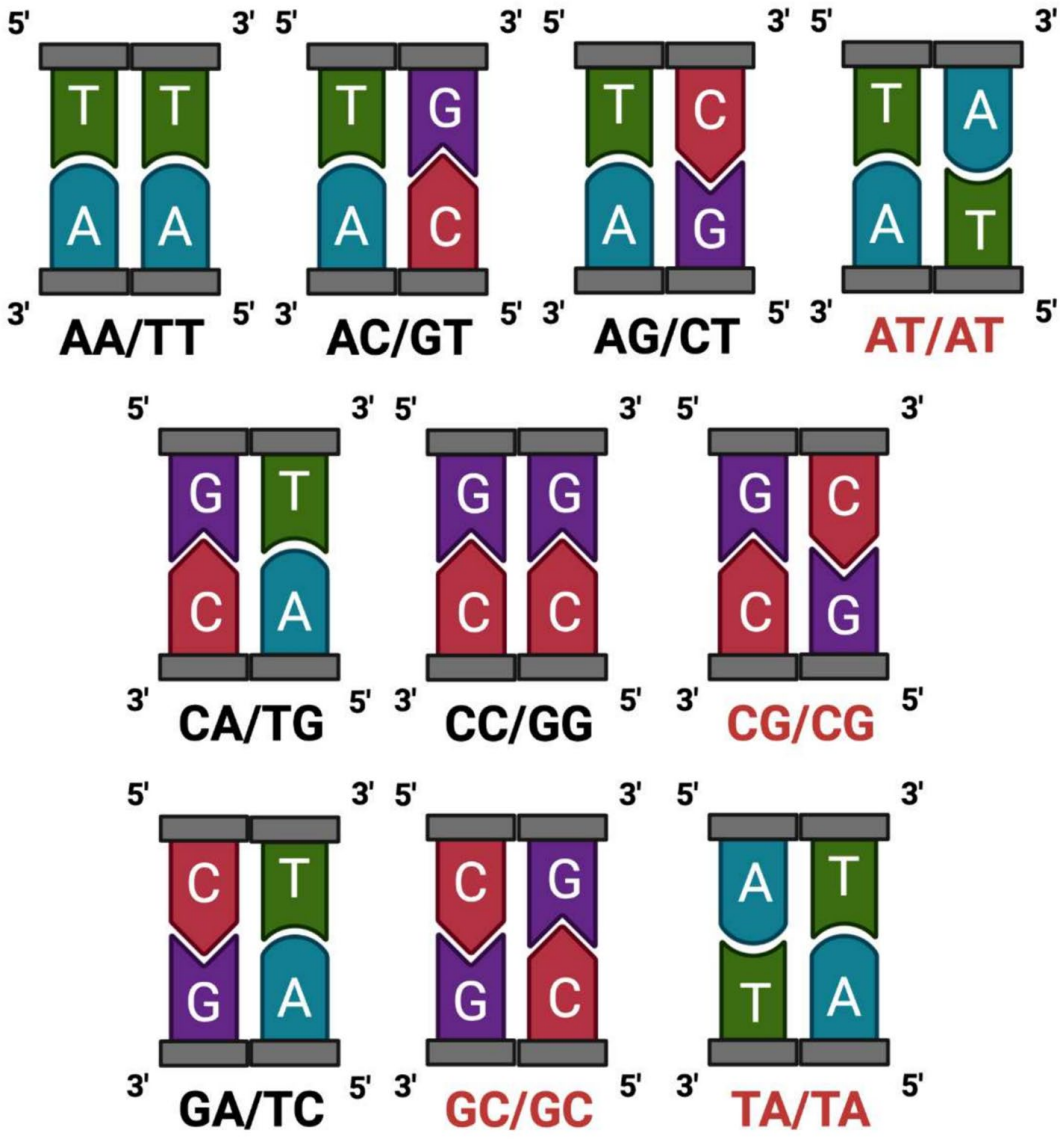
All possible 2-mers and their complements, the palindrome strings are colored red

Due to the fact that a DNA sequence consists of two strings, one of which is the complement of the other, we group all 2-mers into ten classes, $T_i$, $1\le i\le 10$, of 2-mers and their complements. Four 2-mers AT, CG, GC, and TA are palindromes (their complements are equal to themselves). Therefore six classes are of size two, and four are of size 1 (Fig. 1). This study proposes a vector for calculating the repetition frequency of substrings of length between 2 and 10 using only their initial and terminal nucleotides. A substring of size *l* is in class $T_i$ if the 2-mer generated by its initial and terminal nucleotides belongs to $T_i$. For a contig Z, we consider all its substrings of size *l*, $2\le k\le 10$. Let $a_{ki}$ denote the number of substrings of size *l* which belong to bin $T_i$. AFIT vector corresponds to contig Z defined as follows:

$$\begin{aligned} AFIT_Z = (a_{2,1},a_{2,2},..., a_{10,10}) \end{aligned}$$

By this method, we aggregate a contig’s *l*-mer frequencies based on their initial and terminal nucleotides to a vector (AFIT vector) of size 90.

The rationale for this approach is to overcome limitations seen in traditional methods that rely on k-mer frequencies when binning metagenomic data. Typically, the short sizes of reads and contigs provided for metagenomic problems create constraints when utilizing k-mers. The utilization of k-mer frequencies to construct a composition vector results in an enlarged vector size, particularly noticeable with larger k-mers, leading to sparsity in the composition vector for each contig. Specifically, in metagenomic data analysis, numerous k-mers may be absent in a given contig or might exist with very low frequencies. Consequently, these zero or low-frequency components may not significantly contribute to the overall pattern or composition of the DNA sequence. To tackle this challenge, the AFIT approach aggregates k-mer information into equivalent classes based on their initial and terminal nucleotides. Its primary objective is to address the abundance of zero or low-frequency components within the vector. Consequently, reducing sparsity and the overall size of the composition vector results in a more appropriate representation. Additionally, the AFIT vector encapsulates detailed information concerning substring compositions ranging from 2 to 10 nucleotides. By emphasizing similar information through grouping, its purpose is to enhance the representation and comprehension of the structural characteristics and composition of the DNA sequence. It is notable to mention that a similar approach was used for the prediction of mRNA sub-cellular localization [27], showing that the proposed AFIT vector provides useful information on DNA sequences.

### Model setup

**Fig. 2:**
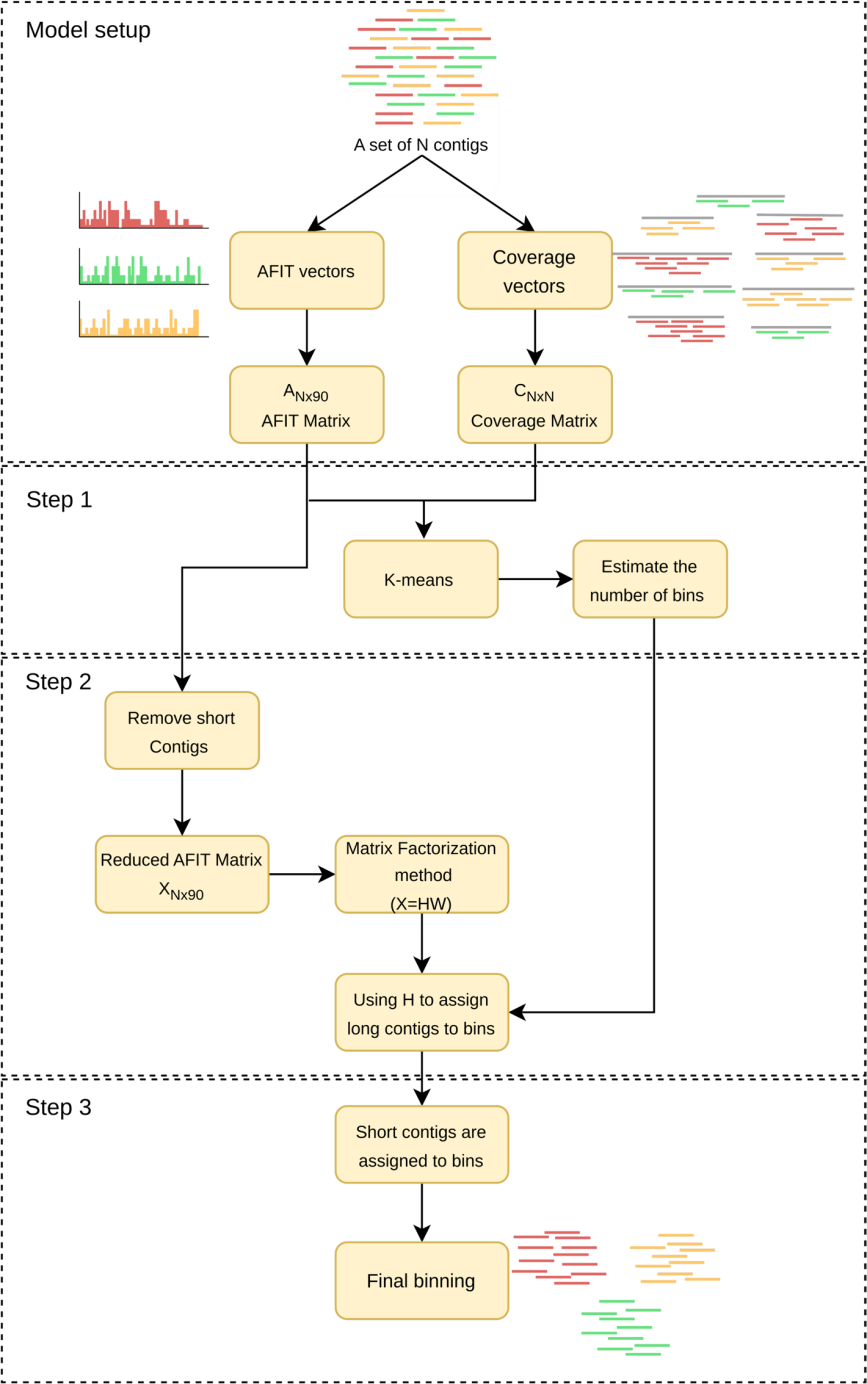
The overview of AFITBin pipeline. This figure shows a visual representation of AFITBin’s workflow, highlighting key steps involved in the process of contig binning. First, the AFIT Matrix and Coverage Matrix are built. The number of bins is determined using the K-means algorithm. The short contigs are set aside, and the bins are created. The short contigs are then assigned to their bins and the final binning is given

In this section, we discuss AFITBin’s approach to the metagenomic contig binning challenge. As previously stated, most binning methods depend on similarity metrics between contigs based on *l*-mers frequency distributions. In this paper, we presented a new approach for generating the composition vector that outperforms prior methods based on *l*-mer counts, such as the TNF vector. Figure 2 depicts the AFITBin processing pipeline. Each step will be explained in detail in the following subsections. AFITBin utilizes two distinct genomic features, the AFIT vector and the coverage distance of contigs, in order to obtain the genomic bins. This is achieved through matrix factorization and solving an optimization problem. In two phases, this algorithm assigns contigs to their predetermined bins. First, contigs shorter than a predetermined length threshold are set aside, while the remaining contigs are assigned to bins based on the methodology explained in this section. Subsequently, the shorter contigs are assigned to their respective bins using a slightly different approach.

Let *N* contigs exist. In this step, the composition matrix is constructed, with each column representing the 90-dimensional AFIT vector obtained in the previous section. The dimension of this feature matrix is $N\times 90$, represented in this paper as $A_{N\times 90}$. The coverage distance between two contigs is calculated using the mean coverage and variance of coverage information for each contig. It is assumed that the distribution of this data is approximately normal. Similar to [28], we consider the non-shared area under the normal distribution graphs of the two contigs to be their coverage distance. As a result, we build a symmetric coverage matrix $C_{N\times N}=[c_{ij}]$, where $c_{ij}$ represents the coverage distances between contigs *i* and *j*. AFITBin consists of three steps:

#### Step1: estimate the number of OTUs

The number of OTUs (bins) is a required input for AFITBin. In this proposed method for determining the number of OTUs in a dataset, similar to the method presented in [29], the k-means algorithm [30] is initialized with a small number of bins, and k is increased until at least 40 percent of the bins remain empty. To use the k-means algorithm, a distance between contigs is required. The distance between two contigs *i* and *j* is defined as $\frac{1}{2}(d_{i,j}+c_{i,j})$ where $d_{i,j}$ denoted the Euclidian distance between the *i*th and *j*th rows of matrix *A*.

#### Step 2: obtaining composition binning index and contig affiliation matrix

The rows and columns corresponding to contigs smaller than a threshold size are removed from matrices *A* and *C* to obtain the reduced matrices *X* and *Y* with *m* rows, to prevent errors caused by insufficient compositional information. We determined the threshold to be 1200 bp such methods used in [31, 32]. Assume that input contigs are associated with k distinct bins, which is obtained in the previous step. Using formula 1, we want to factor *X* into two matrices, $H_{m\times k}=[h_{ij}]$ and $W_{k\times 90}$, where *W* represents the composition index for each bin and *H* represents a contig belonging to a bin. As a result, each row of *H* is a One-hot vector. If contig *i* belongs to bin *j*, $h_{ij}=1$; otherwise, $h_{ij}=0$.

1 $$\begin{aligned} X = H \times W \end{aligned}$$

In order to obtain the matrices *W* and *H*, we solved the subsequent optimization problem shown in formula 2:

2 $$\begin{aligned} \arg \min _{W, H}\Vert X-HW\Vert ^2 \end{aligned}$$

where

$$\begin{aligned} \ H \in \{0, 1\}^{n\times k}, ~and~ \Vert H_n\Vert =1 \end{aligned}$$

where $\Vert \Vert$ denotes the Frobenius norm of a matrix. Consider that the optimization problem is an NP-hard integer programming problem that requires a substantial computational effort to solve. We circumvent the binary restriction of *H* to solve this computational problem. Therefore, the equation is modified to the following minimization problem:

3 $$\begin{aligned} \arg \min _{W, H}\Vert X-HW\Vert ^2 + \alpha \sum _{n=1}^N\Vert H\Vert ^2+\beta \Vert Y*(HH^T)\Vert ^2 \end{aligned}$$

where $H^T$ is the transpose of *H*, and $*$ is regarded as an element-wise multiplication of two matrices, and $\alpha$ and $\beta$ are hyperparameters. This type of matrix multiplication takes into account a binning error based on the coverage distance between two contigs. To solve the second minimization problem, the Conjugate Gradient method is selected, and matrices *W* and *H* are successively optimized at each iteration of this method. After calculating the matrix *H*, the contig *i* is considered to belong to bin *j* if and only if *j* is the bin that $h_{ij}$ is the maximum between all $h_{ir}$, $1\le r \le k$.

#### Step 3: assigning short contigs to bins

In the previous step, short contigs are eliminated, long contigs are binned, and a composition feature vector is assigned to each bin. In this step, each short contig is assigned to one of the obtained bins. To achieve this, for every small contig, v, and bin B we assign two scores. Let *W*(*B*) denote the row of *W* correspond to the bin B. The first score is defined as:

$$\begin{aligned} S_1(B,v)=d(W(B), AFIT_v). \end{aligned}$$

The second score is defined as:

$$\begin{aligned} S_2(B,v)=\frac{\sum _{b\in B} d(Y(v),Y(b))}{|B|} \end{aligned}$$

where b is a contige in B and d is Euclidean distances. Now, the contig v is assigned to the bin B(v) if:

$$\begin{aligned} S_1(B(v),v)+S_2(B(v),v)=\min \{S_1(B,v)+S_2(B,v)| \text {B is between the obtained bins}\}. \end{aligned}$$

### Datasets

In this section, we will briefly describe the datasets used to evaluate our method in this paper. The Sharon dataset [33] is a real dataset utilized for evaluating various metagenomic analyses. This dataset consists of 18 feces samples collected from a newborn infant at eleven distinct intervals. These samples were sequenced on an ILLUMINA machine, and the resulting reads are accessible in the *NCBI Sequence ReadArchive* database with the accession number SRA052203. The researchers who gathered this dataset assembled the reads into 2,329 contigs and, after analyzing the contigs, assigned them to 33 distinct microbial species. The UC Berkeley Genetic Information Database contains these contigs and identified variants (https://ggkbase.berkeley.edu/carrol/organisms).

Another dataset used to evaluate metagenomic binning methods is the CAMI [34] challenge dataset. Diverse datasets of varying complexity have been collected for this challenge to evaluate various metagenomic tools and analysis techniques. The three datasets used in this study, CAMI-Low, CAMI-Medium, and CAMI-High, contain one sample, two samples, and five samples, respectively. The public can access these datasets via the CAMI Challenge website (https://data.cami-challenge.org/participate).

As a simulated dataset, Strain and Species were considered in this paper, which were simulated by the authors of CONCOCT [19] using a microbial community from the Human Microbiome Project. The authors assembled contigs using reads that were analyzed in various Human Microbiome Project samples [35]. The coverage sequences were then constructed by comparing the constructed contigs to the reads.

### Evaluation criteria

We utilize the precision, recall, and F-score metrics to assess AFITBin’s performance. Precision evaluates the accuracy of the classification, whereas recall evaluates its completeness. Therefore, the F-score, which is the harmonic mean of precision and recall, can be utilized to evaluate the performance of binning methods [36].

Assume *n* is the number of species in a metagenomic dataset and k is the number of bins returned by the binning method. The matrix $M_{k\times n}=[m_{ij}]$ is defined in this case so that the array $m_{ij}$ represents the number of contigs associated with species *i* that are positioned in bin *j* using the binning method. The mathematical expression of these scales defines as follows:

4 $$\begin{aligned} \text {Precision}= & {} \frac{\sum _{i=1}^k\ \max _j m_{ij}}{\sum _{i=1}^k\sum _{j=1}^n m_{ij}} \end{aligned}$$

5 $$\begin{aligned} \text {Recall}= & {} \frac{\sum _{j=1}^n\ \max _i m_{ij}}{\sum _{i=1}^k\sum _{j=1}^n m_{ij}} \end{aligned}$$

6 $$\begin{aligned} \text {F-score}= & {} \frac{2*\text {Precision}*\text {Recall}}{\text {Precision}+\text {Recall}} \end{aligned}$$

## Results

AFITBin is compared to MaxBin 2.0, MetaBat 2, CONCOCT, MetaCon, SolidBin, BusyBee Web, and MetaBinner, which are all described in the previous sections. The parameters $\alpha$ and $\beta$, described in the previous section, are set to 2 and $\frac{3}{4}$ respectively, and the number of bins for AFITBin for each dataset is as shown in Table 1.

**Table 1:** This table shows the number of bins for datasets Strain, Species, Sharon, CAMI-Low, CAMI-Medium, and CAMI-High for AFITBin

	Strain	Species	Sharon	CAMI-Low	CAMI-Medium	CAMI-High
Number of Bins in AFITBin	16	88	25	22	54	103

### Performance on simulated datasets

First, we compare the performance of AFITBin on the simulated datasets Strain and Species to the aforementioned algorithms. The Strain contains 9417, contigs related to 20 distinct microorganisms, which were assembled from the sequenced reads of 64 separate samples.

As shown in Table 2, AFITBin has the highest F-score for the Strain dataset, 0.91, compared to other methods. The second-highest reported F-score is 0.90, which belongs to MetaCon. The highest precision belongs to AFITBin which is 0.92, while its recall is 0.91, which is the second highest recall.

The results of AFITBin are then compared to those of other methods on the Species dataset. The sequenced reads from 64 different samples were used to put together the 37,628 contigs that belong to 101 different microorganisms in the Species dataset. In this dataset, similar to the previous one, the best F-score and precision belong to AFITBin, while its recall is the second best. For both datasets, we saw an increase in the F-score of at least 2 percent, which is a considerable improvement. It is especially promising because other methods have already reached a high F-score near the maximum, indicating that even minor improvements have significant value.

### Performance on the Sharon dataset

In addition, AFITBin is assessed using the Sharon dataset. Sharon is a real dataset from a well-studied microbial experiment in which the species involved have been thoroughly examined, as explained previously. The sequenced reads of 18 distinct samples were used to assemble 2,329 contigs associated with 33 distinct microorganisms. As depicted in Table 2, AFITBin performs comparably to other methods with the best performance for the Sharon dataset across all evaluation criteria. On this dataset, AFITBin and MetaCon have the highest F-score of 0.82.

### Performance on the CAMI datasets

AFITBin is evaluated further on CAMI datasets of varying complexity. As shown in Table 2, AFITBin achieves better binning results than other methods on CAMI-Low, and CAMI-Medium, The CAMI-Low dataset contains 1949 contigs from 40 distinct microbes. The reads from a single sample were used to assemble the contigs in this dataset. Even though AFITBin does not report the highest precision, AFITBin increased recall from 0.48 to 0.59, as shown in Table 2. Evidently, AFITBin obtains the highest F-score compared to all other methods and increased the F-score from 0.56 to 0.62.

CAMI-Medium is made up of 63,447 contigs from 132 different microorganisms. These contigs were assembled from sequenced reads from two different samples. Table 2 demonstrates that AFITBin classifies the contigs of this dataset with a higher F-score than other methods. We were successful in increasing the F-score from 0.42 to 0.43. CAMI-High is made up of 42,038 contigs that represent 132 different types of microorganisms. It was made by putting together the sequenced reads of five samples. AFITBin outperforms the other classification methods in terms of recall and has an F-score of 0.44, which is greater than the F-scores of MetaCon, CONCOCT, MaxBin 2.0, and BusyBee Web, but not MetaBat 2, SolidBin and MetaBinner.

**Table 2:** The overall performances of MaxBin 2.0 (M-Bin2), MetaBat 2 (M-Bat2), CONCOCT (CCT), MetaCon (M-Con), SolidBin (S-Bin), BusyBee Web (B-Bee), MetaBinner (Mt-Bin), and AFITBin (A-Bin) based on precision, recall, and F-score, on real and simulated datasets

	Method	M-Bin2	M-Bat2	CCT	M-Con	S-Bin	B-Bee	Mt-Bin	A-Bin
**Str**	Precision	0.71	0.88	0.87	0.91	**0.92**	0.91	0.70	**0.92**
	Recall	0.81	0.79	**0.92**	0.90	0.83	0.85	0.87	0.91
	F-score	0.75	0.83	0.89	0.90	0.87	0.87	0.77	**0.91**
**Spc**	Precision	0.90	0.93	0.83	0.94	0.95	0.94	0.91	**0.95**
	Recall	0.92	0.91	**0.97**	0.92	0.91	0.87	0.95	0.94
	F-score	0.91	0.92	0.89	0.93	0.92	0.90	0.92	**0.95**
**Sh**	Precision	0.77	**0.82**	0.79	**0.82**	0.79	0.80	0.75	**0.82**
	Recall	0.75	0.80	0.82	**0.83**	0.73	0.69	0.80	**0.83**
	F-score	0.76	0.81	0.81	**0.82**	0.75	0.74	0.77	**0.82**
**C-L**	Precision	0.48	0.45	0.49	0.61	0.80	**0.95**	0.44	0.66
	Recall	0.43	0.44	0.43	0.48	0.44	0.15	0.50	**0.59**
	F-score	0.45	0.45	0.46	0.53	0.56	0.25	0.46	**0.62**
**C-M**	Precision	0.43	0.26	0.34	0.44	0.46	**0.54**	0.38	0.45
	Recall	0.41	0.29	0.39	0.32	0.39	0.32	**0.42**	0.40
	F-score	0.42	0.27	0.37	0.37	0.42	0.40	0.39	**0.43**
**C-H**	Precision	0.39	0.51	0.24	0.33	0.67	**0.85**	0.52	0.34
	Recall	0.40	0.58	0.58	0.56	0.51	0.21	**0.59**	**0.59**
	F-score	0.40	0.54	0.34	0.42	**0.57**	0.33	0.55	0.44

These datasets consist of Strain (Str), Species (Spc), Sharon (Sh), CAMI-Low (C-L), CAMI-Medium (C-M), and CAMI-High (C-H). For each criteria on a specific dataset, the best result among all methods are shown in bold font. As shown in the table, AFITBin outperforms all other methods on all datasets based on F-score, except CAMI-High, which means AFITBin has the best overall performance on the constructed bins. However, on the CAMI-High dataset, AFITBin has the highest recall among other methods, showing the completeness of the constructed bins

While AFITBin demonstrates precision values below the best precision obtained by other methods in certain datasets, it’s crucial to recognize that in these datasets, the methods exhibiting high precision often display very low recall. For instance, BusyBee Web, despite showcasing a precision of 0.85 on the CAMI-High dataset, notably suffers from a substantially low recall of 0.21. This lower recall implies that BusyBee Web generates a significantly smaller number of accurate bins compared to the expected number, resulting in a low F-score. When assessing algorithm performance, solely focusing on precision or recall might not offer a comprehensive understanding. The F-score, recognized as the harmonic mean of precision and recall, provides a more balanced evaluation. Moreover, no single method can consistently outperform all others across every dataset. However, as depicted in Table 2, AFITBin exhibits superior overall performance compared to the other methods. Although our method did not achieve the best result on the CAMI-High dataset, AFITBin performed the best among the five remaining datasets. The modest yet consistent improvement demonstrated by AFITBin in this manuscript holds significant importance, particularly considering the complexity of the metagenomic binning challenge. To discuss more about the obtained results, we use CheckM [37], which is a powerful tool widely used in metagenomics to assess the quality of microbial genomes reconstructed from metagenomic data. It evaluates the completeness and contamination levels of genomes, providing valuable insights into the reliability of genomic bins. The results obtained from CheckM analysis revealed crucial information about the quality of the genomic bins produced by both methods. Table 3 shows the performance of two binning methods, AFITBin and MetaBinner using CheckM. AFITBin exhibited promising performance, yielding genomic bins with high completeness levels and low contamination rates. MetaBinner’s performance, as indicated by CheckM results, demonstrated slightly lower completeness levels and marginally higher contamination rates compared to AFITBin. It is noteworthy that in [25], MetaBinner was showcased to surpass all other methods when assessed using CheckM. In conclusion, the CheckM results provide valuable insights into the performance of AFITBin and MetaBinner, highlighting AFITBin’s potential as a robust binning method.

**Table 3:** Comparison of AFITBin and MetaBinner using CheckM on the CAMI-Low dataset

Dataset	Methods	Metrics					
		#bins ($>50$% comp $<10$% cont)	#bins ($>70$% comp $<10$% cont)	#bins ($>90$% comp $<10$% cont)	#bins ($>50$% comp $<5$% cont)	#bins ($>70$% comp $<5$% cont)	#bins ($>90$% comp $<5$% cont)
CAMI-Low	MetaBinner	16.7	14.9	11.3	15.4	13.9	10.6
	AFITBin	**20.0**	**17.5**	**12.2**	**19.1**	**17.2**	**11.9**

For each criteria on a specific dataset, the best result among all methods are shown in bold font

### Comparison of AFIT and TNF vectors

**Fig. 3:**
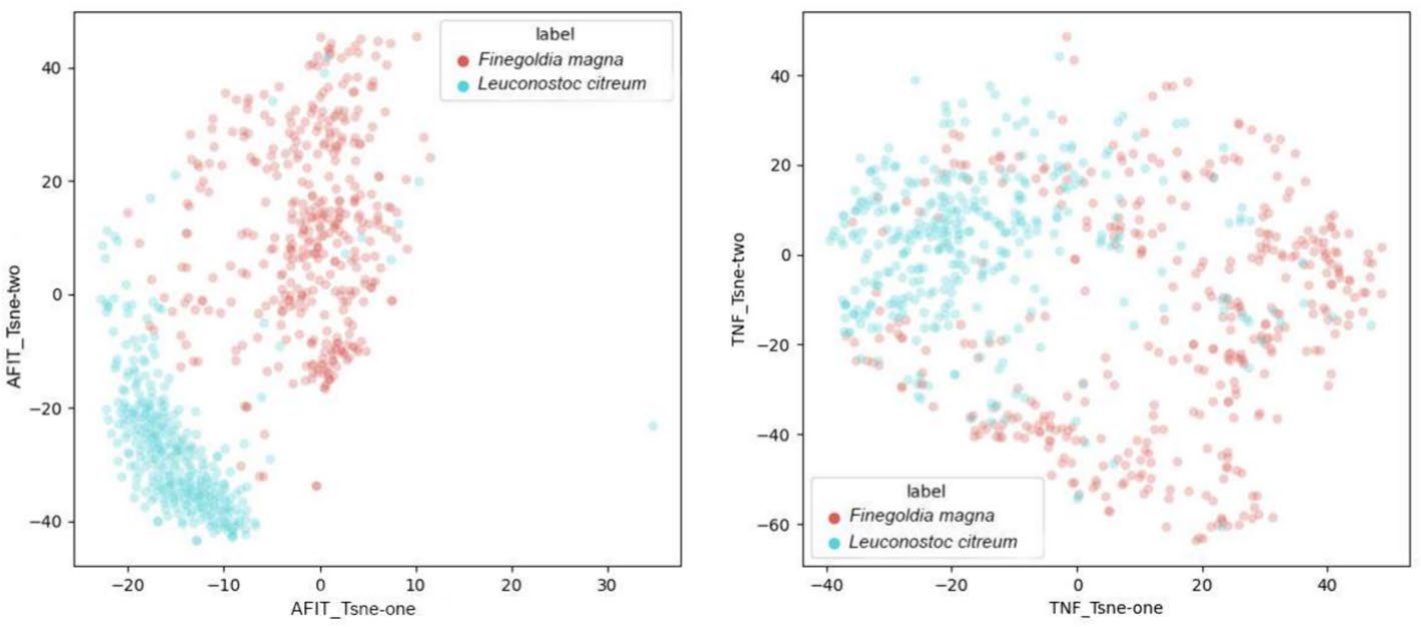
Comparison of the ability of the AFIT vector and TNF vector in clustering two species, *Finegoldia magna* and *Leuconostoc citerum*. The figure on the right depicts the performance of the TNF vector, while the figure on the left depicts the performance of the AFIT vector using the t-SNE algorithm. The pink dots represent contigs from the species *Finegoldia magna*, while the blue dots represent contigs from the species *Leuconostoc citerum*

To determine the significance of the AFIT vector, we considered several distinct strategies. We begin by comparing TNF and AFIT vectors using contigs from species in the Sharon dataset. For each species, we construct AFIT and TNF vectors based on their contigs. Next, we select a pair of species and consider the AFIT and TNF vectors of these chosen species. Then, utilizing the t-Distributed Stochastic Neighbor Embedding (t-SNE) [38] algorithm, these contigs are depicted in two-dimensional space once based on TNF and once based on AFIT. t-SNE is a dimensionality reduction method with the main purpose of visualizing high-dimensional data in a lower-dimensional space, often in two or three dimensions. It is particularly effective for exploring and understanding patterns in complex datasets. The primary goal of t-SNE is to reduce the dimensionality of data points while maintaining their pairwise similarities or distances as much as possible. It accomplishes this by converting the similarities between data points in the high-dimensional space into conditional probabilities, where similar points have higher probabilities of being picked as neighbors. Our findings demonstrate that contigs from the same species can be effectively grouped together using AFIT vectors but not TNF vectors. Figure 3 is an illustration of the outcomes of the t-SNE algorithm for two species, *Finegoldia magna* and *Leuconostoc citerum*, using AFIT and TNF vectors. This figure demonstrates that contigs belonging to the same species can be clustered exceptionally well with AFIT vectors but not with TNF vectors. In the supplementary Figs. S1–S9, we compare the performance of the AFIT vector and TNF vector in clustering two different species, where the right figure shows the performance of the TNF vector and the left figure shows the performance of the AFIT vector for different pairs of species. Our approach and findings align with similar methodologies [39], supporting the effectiveness of using t-SNE to compare AFIT and TNF vectors for genetic characteristics across different species pairs in metagenomic studies.

Recent binning methods have little direct application of Euclidean distance. In the second approach, for additional evaluation, we compare the Euclidean distance obtained by the AFIT vector and the TNF vector for contigs of the same species (intra) and contigs from different species (inter). For this comparison, the Euclidean distance was calculated once between AFIT vectors and once between TNF vectors for more than 2,000 contigs from 33 different species. Table 4 displays the mean and standard deviation of the distances between contigs of the same species and contigs of different species using the AFIT and TNF vectors. This table demonstrates that, compared to the TNF vector, the AFIT vector is better able to differentiate between contigs of the same and different species.

**Table 4:** The mean($\mu$) and variance($\sigma$) of Euclidian distance of both AFIT vector and TNF vector for contigs from the same species (intra) and contigs from different species (inter)

	$\mu _{inter}$	$\sigma _{inter}$	$\mu _{intra}$	$\sigma _{intra}$
Euclidian distance AFIT	0.13	0.1	0.25	0.1
Euclidian distance TNF	0.42	0.2	0.55	0.2

To further assess the effectiveness of the AFIT vector, we conducted distinct evaluations following the methodology outlined by Zhou et al. [40], barcodes of several genomes are plotted using the AFIT and TNF vectors.

**Fig. 4:**
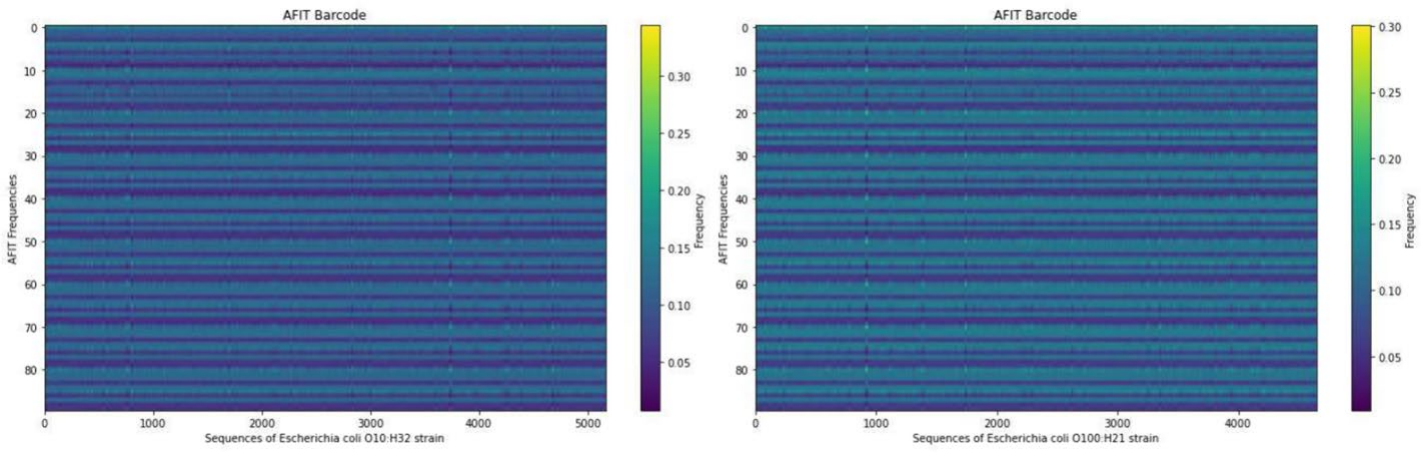
Comparison of the barcode of AFIT vector on the two strains, *E. coli O10:H32* and *E. coli O100:H21*. The figure on the right depicts the barcode of *E. coli O100:H21* genome, while the figure on the left depicts the barcode of *E. coli O10:H32* genome plotted using the AFIT vector. The barcode of E. coli strains using AFIT vector reveals remarkable genome-wide similarity and consistency

We investigated the AFIT vector’s consistency across bacterial genomes. Our analysis revealed notable consistency in frequency patterns from the start to the end of bacterial genomes, even when comparing strains from the same species. Specifically, we focused on plotting barcodes using the AFIT vector for two strains of *Escherichia coli (E.coli)* bacteria: *E. coli O10:H32* and *E. coli O100:H21*. The resulting plots shown in Fig. 4 indicated remarkable consistency throughout the entire genomes. Additionally, the barcodes generated using AFIT for both strains exhibited striking similarity and provided informative insights.

**Fig. 5:**
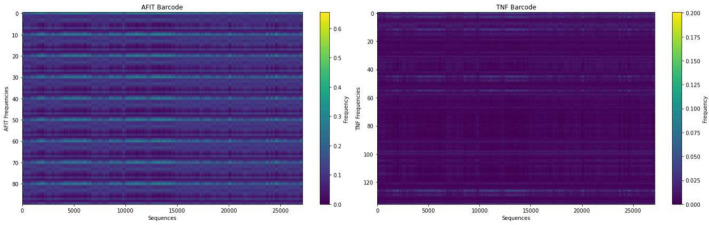
Comparison of the barcodes of the AFIT and TNF vector on *Apis cerana*. The figure on the right shows the performance of the TNF vector, while the figure on the left shows the performance of the AFIT vector. TNF barcode lacked diversity with values mostly near zero, while AFIT barcode showed consistent yet distinct values across components, highlighting AFIT’s effectiveness in capturing unique genome traits

Furthermore, we extended our evaluation to the genome of *Apis cerana*, generating its barcode using both the AFIT and TNF vectors. Our observations shown in Fig. 5 illustrated that the barcode derived from TNF vectors lacked informativeness due to nearly identical values across all components, predominantly near zero. Conversely, the barcode created with the AFIT vector offered significant information. It demonstrated consistency across each component while highlighting discernible differences between the values of various components. This emphasized the utility and effectiveness of the AFIT vector in capturing distinctive genome characteristics.

## Discussion

Binning metagenomic contigs is a crucial step in metagenomic studies. Metagenomic binning is the process of grouping reads or contigs and assigning them to specific species. Using features extracted from read or contig sequences, binning methods attempt to group these sequences. Typically, these features are compositional features, coverage features, or both. In this paper, we introduced a novel composition vector, AFIT, and the AFITBin binning method, which utilizes both AFIT vector and coverage data. To evaluate the significance of the new composition vector, we not only investigate the performance of AFITBin by comparing its results on different datasets with those of well-known methods, but we also demonstrate how well this vector distinguishes contigs of different species compared to an earlier method.

In addition, we evaluated the performance of various cutting-edge binning algorithms on both simulated and real datasets. Using the composition feature vector, AFIT, AFITBin improved its performance in terms of precision, recall, and F-score. As the results demonstrate, AFITBin outperformed the majority of the stated methods on most of the datasets. According to the findings of this investigation, neither of the introduced methods performs optimally on every dataset. On some datasets, AFITBin has the best performance, whereas on others, it performs similarly to the method with the best-reported results. Aside from this, the new proposed vector has smaller dimensions than the old vector, reducing the computational costs of the binning algorithm and the complexity of designing an appropriate binning algorithm. The AFIT vector introduced by this method can be largely responsible for this outcome.

## Conclusion

We demonstrate that the incorporation of the AFIT vector into our binning algorithm allows AFITBin to accommodate datasets with diverse characteristics. In addition, the AFIT vector combines information from previous oligonucleotide (*l*-mer) frequency feature vectors, making it superior due to its enhanced performance. Clearly, there is still a substantial amount of work to be done to improve metagenomic binning. As previously stated, the majority of binning methods fall short when it comes to determining the actual number of species to be binned. AFITBin currently uses the k-means clustering algorithm to predict the number of species, but errors can lead to incorrectly binned contigs and reduce the precision of the final binning result. Consequently, the development of a suitable method for predicting the actual number of categories is one of the fields that can be utilized to develop the paper’s solutions.

### Supplementary Information

Supplementary Material 1

## Data Availability

The code of AFITBin is freely available at: https://github.com/SayehSobhani/AFITBin. The Sharon dataset (accession number SRA052203) is available at UC Berkeley’s Genetic Information Database (https://ggkbase.berkeley.edu/carrol/organisms). The CAMI challenge dataset including CAMI-Low, CAMI-Medium, and CAMI-High datasets, is accessible through the CAMI Challenge website (https://data.cami-challenge.org/participate). Also the *E. coli O10:H32* (GCF_013282315.1) and *E. coli O100:H21* (GCF_015571795.1) dataset of genomes is available at NCBI.
