# Supplementary material for: AFITbin: a metagenomic contig binning method using aggregate l-mer frequency based on initial and terminal nucleotides
Source: BMC Bioinformatics. 2024 Jul 16;25:241. doi: 10.1186/s12859-024-05859-7 (PMC11253361; doi:10.1186/s12859-024-05859-7)
Supplement: Supplementary file 1 — Supplementary Material 1 [file 12859_2024_5859_MOESM1_ESM.pdf]

AFITBin: A metagenomic contig binning method using aggregate l-mer frequency based on initial and terminal nucleotides

Amin Darabi[1], **Sayeh Sobhani**[2,1], **Rosa Aghdam**[3,2], **and Changz Eslahchi**[1,2]

[1] *Department of Computer and Data Sciences, Faculty of Mathematical Sciences, Shahid Beheshti University, Tehran, Iran*
[2] *School of Biological Sciences, Institute for Research in Fundamental Sciences (IPM), Tehran, Iran*
[3] *Wisconsin Institute for Discovery, University of Wisconsin-Madison, Madison, WI 53715, USA*

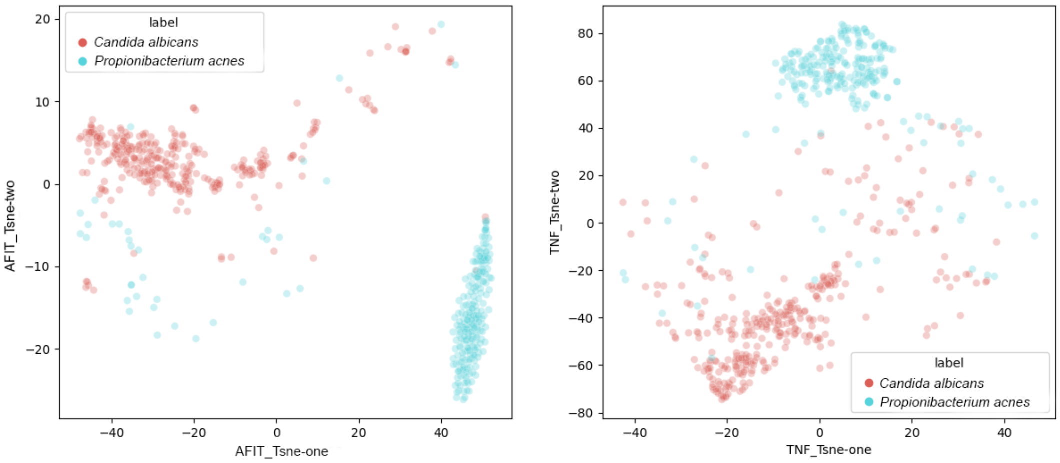

Fig. S1. Comparison of the ability of the AFIT vector and TNF vector in clustering two species, *Candida albican*, and *Propionibacterium acnes*. The figure on the right depicts the performance of the TNF vector, while the figure on the left depicts the performance of the AFIT vector using the t-SNE algorithm. The pink dots represent contigs from the species *Candida albican*, while the blue dots represent contigs from the species *Propionibacterium acnes*.

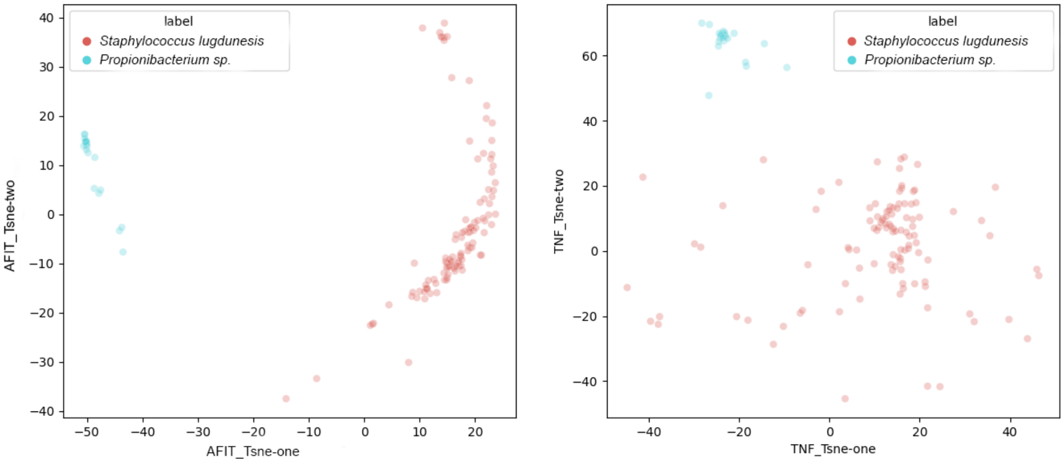

Fig. S2. Comparison of the ability of the AFIT vector and TNF vector in clustering two species, *Staphylococcus lugdunensis*, and *Propionibacterium sp*. The figure on the right depicts the performance of the TNF vector, while the figure on the left depicts the performance of the AFIT vector using the t-SNE algorithm. The pink dots represent contigs from the species *Staphylococcus lugdunensis*, while the blue dots represent contigs from the species *Propionibacterium sp*.

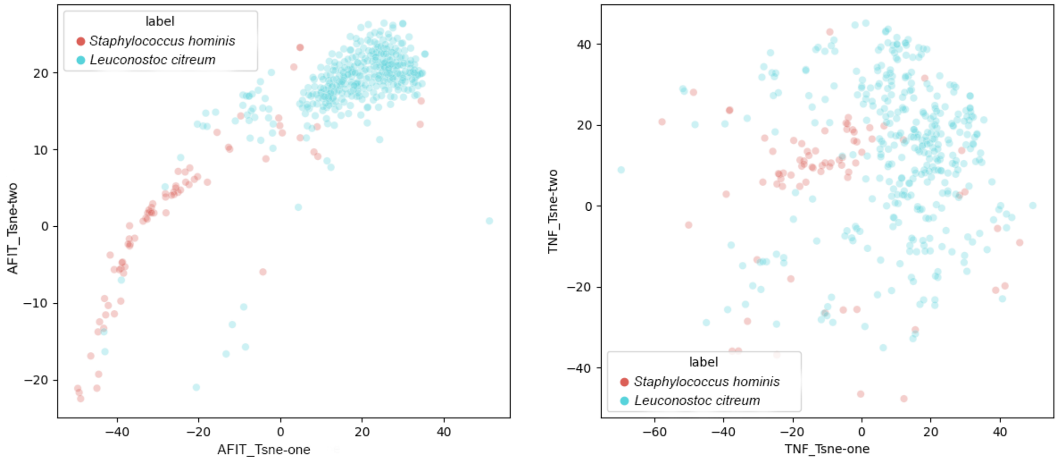

Fig. S3. Comparison of the ability of the AFIT vector and TNF vector in clustering two species, *Staphylococcus hominis*, and *Leuconostoc citerum*. The figure on the right depicts the performance of the TNF vector, while the figure on the left depicts the performance of the AFIT vector using the t-SNE algorithm. The pink dots represent contigs from the species *Staphylococcus hominis*, while the blue dots represent contigs from the species *Leuconostoc citerum*.

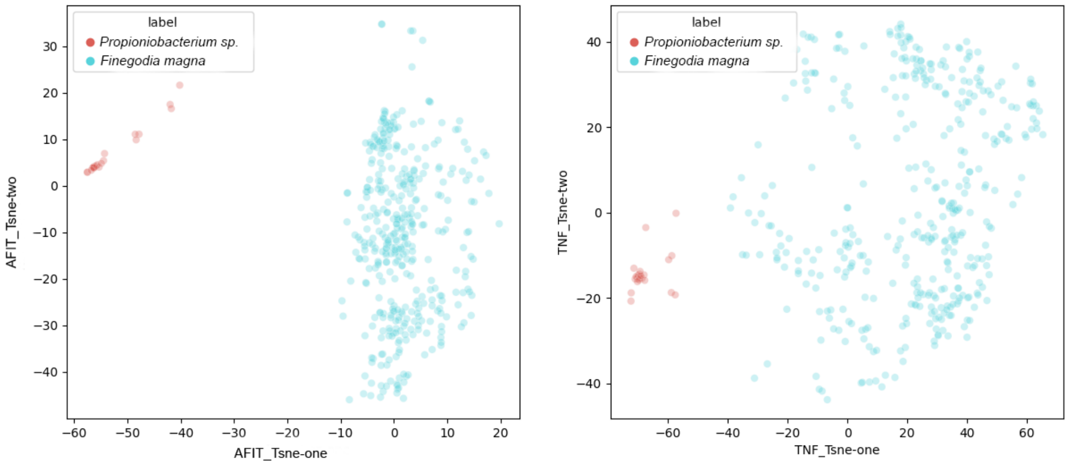

Fig. S4. Comparison of the ability of the AFIT vector and TNF vector in clustering two species, *Propionibacterium sp* and *Finegoldia magna*. The figure on the right depicts the performance of the TNF vector, while the figure on the left depicts the performance of the AFIT vector using the t-SNE algorithm. The pink dots represent contigs from the species *Propionibacterium sp*, while the blue dots represent contigs from the species *Finegoldia magna*.

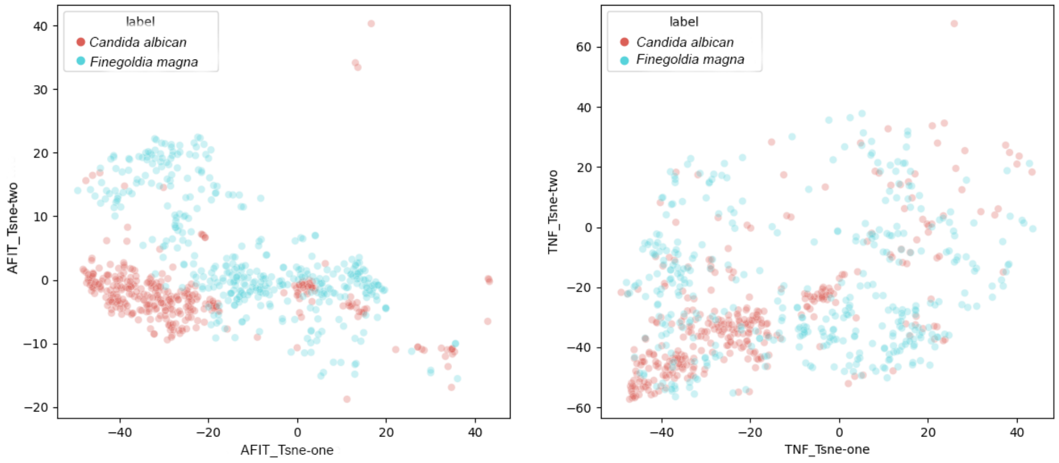

Fig. S5. Comparison of the ability of the AFIT vector and TNF vector in clustering two species, *Candida albican* and *Finegoldia magna*. The figure on the right depicts the performance of the TNF vector, while the figure on the left depicts the performance of the AFIT vector using the t-SNE algorithm. The pink dots represent contigs from the species *Candida albican*, while the blue dots represent contigs from the species *Finegoldia magna*.

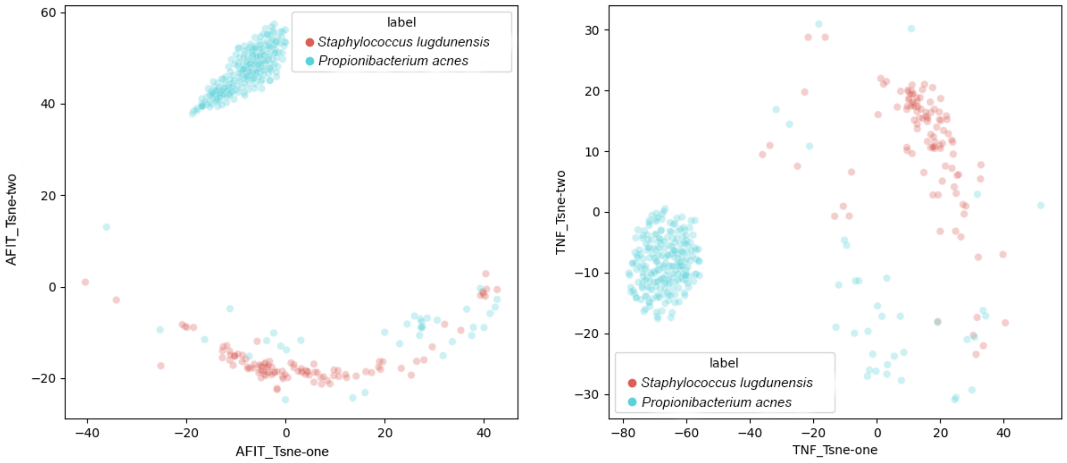

Fig. S6. Comparison of the ability of the AFIT vector and TNF vector in clustering two species, *Staphylococcus lugdunensis* and *Propionibacterium acnes*. The figure on the right depicts the performance of the TNF vector, while the figure on the left depicts the performance of the AFIT vector using the t-SNE algorithm. The pink dots represent contigs from the species *Staphylococcus lugdunensis*, while the blue dots represent contigs from the species *Propionibacterium acnes*.

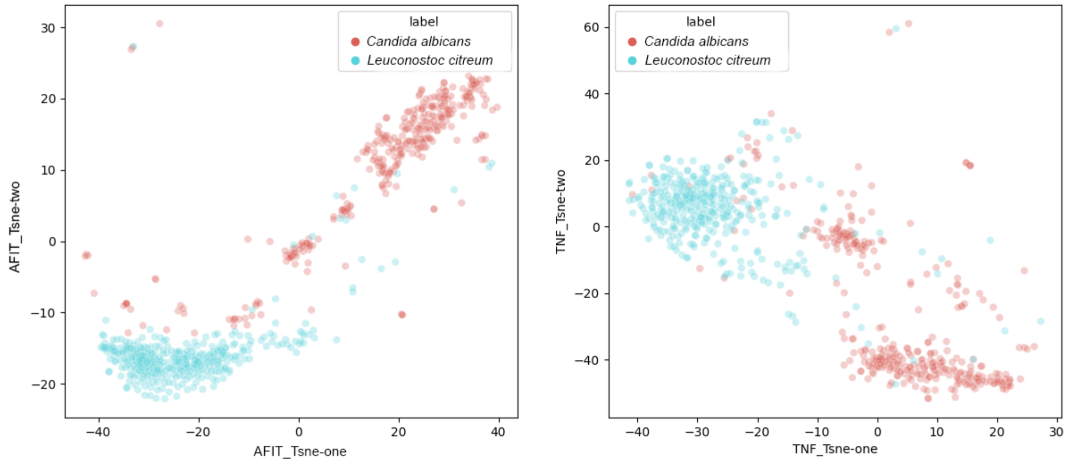

Fig. S7. Comparison of the ability of the AFIT vector and TNF vector in clustering two species, *Candida albican* and *Leuconostoc citerum*. The figure on the right depicts the performance of the TNF vector, while the figure on the left depicts the performance of the AFIT vector using the t-SNE algorithm. The pink dots represent contigs from the species *Candida albican*, while the blue dots represent contigs from the species *Leuconostoc citerum*.

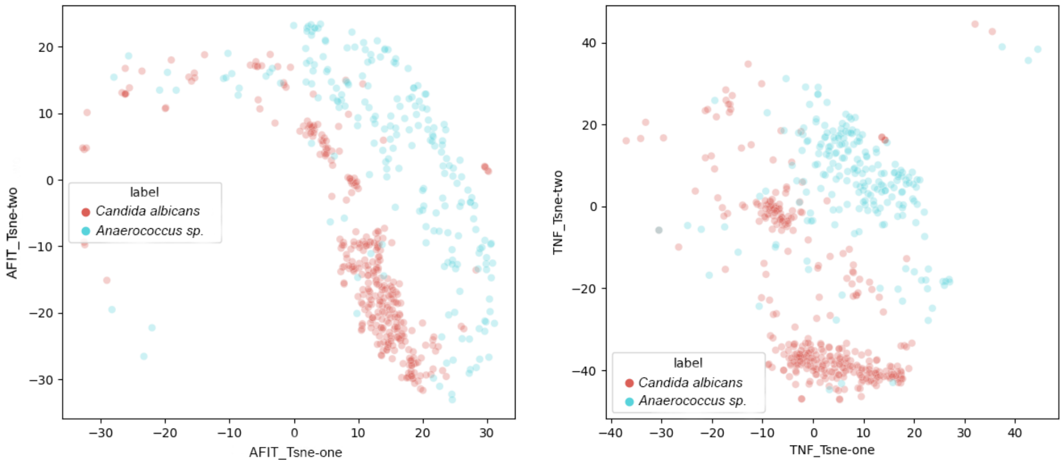

Fig. S8. Comparison of the ability of the AFIT vector and TNF vector in clustering two species, *Candida albican* and *Anaerococcus sp*. The figure on the right depicts the performance of the TNF vector, while the figure on the left depicts the performance of the AFIT vector using the t-SNE algorithm. The pink dots represent contigs from the species *Candida albican*, while the blue dots represent contigs from the species *Anaerococcus sp*.

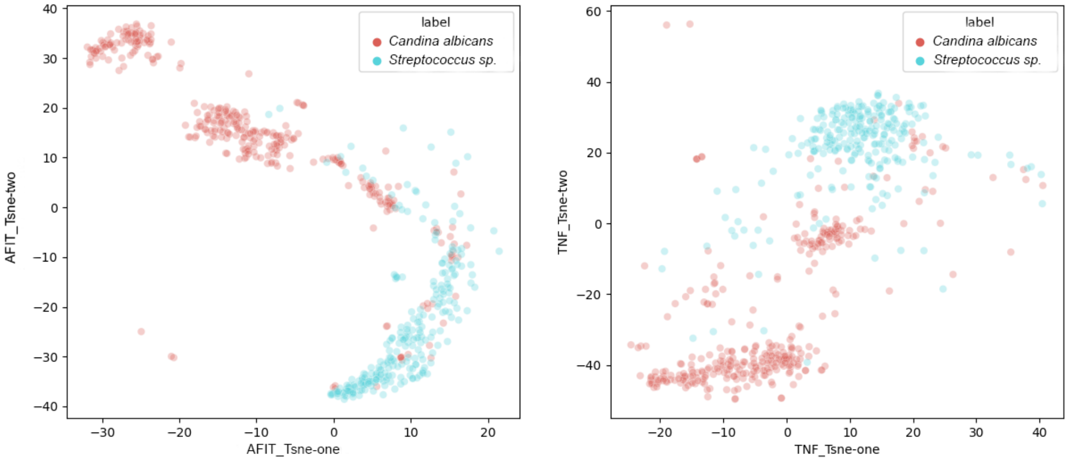

Fig. S9. Comparison of the ability of the AFIT vector and TNF vector in clustering two species, *Candida albican* and *Streptococcus sp*. The figure on the right depicts the performance of the TNF vector, while the figure on the left depicts the performance of the AFIT vector using the t-SNE algorithm. The pink dots represent contigs from the species *Candida albican*, while the blue dots represent contigs from the species *Streptococcus sp*.
